# Rising burden of enterococcal bacteremia in Victoria, Australia: population-based incidence and antimicrobial resistance trends from three decades of surveillance

**DOI:** 10.1128/aac.01526-25

**Published:** 2026-02-04

**Authors:** Marwa Talat Alhothali, Torsten Seemann, Patiyan Andersson, Norelle Sherry, Jeremy D. Silver, Oscar C. Howden, Mathilda Wilmot, Wendy Siryj, Mark G. Veitch, Benjamin P. Howden, Courtney R. Lane

**Affiliations:** 1 Department of Microbiology & Immunology, The University of Melbourne at the Peter Doherty Institute for Infection & Immunity, Melbourne, Victoria, Australia; 2 Centre for Pathogen Genomics, The University of Melbourne, Melbourne, Victoria, Australia; 3 AMR Surveillance and Response Unit, Microbiological Diagnostic Unit Public Health Laboratory, The University of Melbourne at the Peter Doherty Institute for Infection & Immunity, Melbourne, Victoria, Australia; 4 Statistical Consulting Centre, The University of Melbourne, Melbourne, Victoria, Australia; 5 Tasmanian Department of Health, Hobart, Tasmania, Australia; Columbia University Irving Medical Center, New York, New York, USA

**Keywords:** *Enterococcus *species, antimicrobial resistance, vancomycin, bacteremia, inverse probability weighting

## Abstract

Enterococcal bacteremia is a common healthcare-associated infection, associated with significant morbidity and mortality, with the emergence of vancomycin-resistant enterococci further complicating treatment and clinical outcomes. Despite this, long-term estimates of population-based incidence and antimicrobial resistance trends are limited. We aim to describe the burden of enterococcal bacteremia in Victoria, Australia (population 7.0 million), over a 35-year period. We conducted a retrospective analysis of laboratory-confirmed enterococcal bacteremia episodes voluntarily reported to the Victorian Hospital Pathogen Surveillance Scheme database from 1988 to 2022. Population-based incidence was estimated using inverse probability weighting to adjust for inconsistent hospital participation. Incidence per 10,000 hospital admissions was determined for the period 2011–2022. Antimicrobial resistance was calculated as the annual proportion of resistant isolates among all tested isolates. Overall, 11,157 enterococcal bacteremia episodes were identified, mainly *Enterococcus faecalis* (*n* = 6,915, 61.9%) and *Enterococcus faecium* (*n* = 3,558, 31.9%). Incidence increased from <3 episodes/100,000 population in 1988 to >10 by 2022. Incidence per 10,000 hospital admissions within Victoria has also increased from 2.8 in 2011 to 4 in 2022. Although *E. faecalis* remained mostly susceptible to tested antibiotics, *E. faecium* showed persistently high levels of vancomycin resistance, ranging from 50.7% (*n* = 69/136) to 66.5% (*n* = 139/209) over the past decade. Increasing incidence and high rates of vancomycin resistance among *E. faecium* highlight the ongoing clinical and public health challenge posed by enterococcal bacteremia. Applying statistical modeling to account for variability in hospital participation improves the certainty of incidence measures and strengthens the evidence for true increase in disease burden.

## INTRODUCTION

*Enterococcus* species are natural colonizers of the gastrointestinal tract but can cause severe infections including bloodstream infections (BSIs). The most commonly isolated species in nosocomial settings are *Enterococcus faecalis* and *Enterococcus faecium* (1). In the United States, all *Enterococcus* species were ranked the second most common cause of healthcare-associated infections (HAIs) in adults between 2018 and 2021 (2). Similarly, in Europe, *Enterococcus* species were the second most frequently isolated pathogen from intensive care unit (ICU)-acquired BSI for 2019 (3).

Enterococci are intrinsically resistant to many classes of antibiotics, including β-lactams, cephalosporins, aminoglycosides, and lincosamides. *E. faecium* is particularly concerning given its ability to acquire resistance to glycopeptides, including vancomycin, an antibiotic used against infections caused by gram-positive bacteria (4). Vancomycin-resistant *E*. *faecium* (VREfm) has emerged as a major healthcare challenge globally (5).

Vancomycin-resistant *Enterococcus* (VRE) was first reported in the late 1980s in the United Kingdom and Europe, and later the United States (6). In Australia, VRE was first identified in 1994, in Melbourne. Since 1996, there has been a gradual increase in VRE cases around Australia (7). Nowadays, it has spread to many countries (6), with Australia’s rates of VREfm ranking among the highest globally (8). The Australian Group on Antimicrobial Resistance (AGAR) found that in 2021, 40.2% of *E. faecium* bacteremia isolates in Australia exhibited vancomycin resistance, considerably higher than estimates from most European nations, but lower than those reported from the United states (9, 10). Invasive VRE infections are associated with substantial mortality (11), with a 30-day all-cause mortality rate of 28.7% (12). The treatment of invasive VRE infections is particularly challenging, often requiring dual antimicrobial therapy (1).

Locally, Victoria has experienced a substantial burden of VRE, evidenced by multiple studies documenting high prevalence, healthcare-associated outbreaks, and interhospital clonal spread (8, 13–16). Despite that and the clinical implications associated with BSIs, epidemiological reports on the incidence of enterococcal bacteremia at the population level in Victoria are scarce. Although AGAR has reported AMR patterns from enterococcal BSI since 2011, Victorian data are under-represented (12), and incidence estimates are not provided. Similarly, the Victorian healthcare-associated infection surveillance system monitors selected HAI, but not *Enterococcus* species, and reports infection rates using hospital activity metrics such as occupied bed days rather than population-based denominators (17). In contrast, this study presents comprehensive, representative, and long-term data on the burden of enterococcal bacteremia in Victoria, Australia, providing both population-based incidence and antimicrobial resistance trends, generating important insights to inform public health decisions.

## MATERIALS AND METHODS

### Setting

This study was conducted in Victoria, Australia. It is the second most populous state in Australia with an estimated population of approximately 7.0 million in March 2025 (18). The state’s healthcare services are provided through more than 300 public and private hospitals, rural and regional health services, specialist mother and child hospitals, and small and specialist rehabilitation hospitals (19). The Microbiological Diagnostic Unit Public Health Laboratory (MDU PHL) serves as the state’s reference laboratory for testing and typing of bacterial pathogens of public health significance (20).

### Study design, data source, and episode definition

We conducted a retrospective analysis of invasive enterococcal infection isolates from 1988 to 2022, utilizing data from the Victoria Hospital Pathogen Surveillance Scheme (VHPSS) database. The VHPSS is a voluntary surveillance system developed in 1988 by MDU PHL to monitor bloodstream and cerebrospinal fluid infections caused by bacteria or fungi in Victoria. The VHPSS receives data from public, private, metropolitan, and regional laboratories referred from Victorian hospitals. VHPSS coverage of blood culture isolates was previously estimated to range between 60% to 80% (21).

An episode of bacteremia was defined as the first isolation of a species of *Enterococcus* from a blood specimen from a patient within a 14-day period. A new positive blood isolate collected from the same patient and with the same *Enterococcus* species collected more than 14 days after the most recent positive isolate was considered a new episode of bacteremia. Isolates collected within 14 days of the most recent episode were classified as duplicates and excluded as outlined in the exclusion criteria (see Fig. S1).

### Identification and antimicrobial susceptibility testing (AST)

Species identification and phenotypic AST were performed at the submitting laboratory, using Vitek, disc diffusion, *E*-test, and/or microscan according to international standards. AST data were reported as S (sensitive), I (intermediate) or R (resistant), N (No interpretation), and D (dose-dependent) as per the submitting laboratory protocols. For analysis, I, N, D, and S results were re-classified as non-resistant. We collected AST results for ampicillin, amoxicillin, penicillin G, high-level gentamicin, vancomycin, teicoplanin, daptomycin, and linezolid.

### Statistical analysis

Population-based annual incidence per 100,000 was estimated using three approaches: (i) unadjusted (raw), (ii) adjusted by estimated hospital coverage, and (iii) weighted incidence (Supplementary methods E1 to E3, respectively). To address the issue of underreporting due to inconsistent participation, we applied inverted probability weighting (IPW). This statistical approach is commonly used in surveys and epidemiological research to adjust for missingness or selection bias (22, 23). Here, we reweighted episode counts based on the probability of hospital participation. The IPW method incorporated hospital characteristics (peer group and remoteness area) and reporting year to model participation probability. This approach assumes that underreported data are missing and therefore observed episode counts are scaled up to represent the full population. A detailed description of incidence estimation and the IPW methodology is provided in the Supplementary methods.

To account for changes in population age structure over time, we first calculated annual age-specific incidence rates for each 5-year age group using the corresponding estimated resident population for that year. We then applied direct age-standardization using the 2011 estimated resident population of Victoria, as this year corresponds to the midpoint of our study period. For each year, the age-standardized incidence was calculated as the weighted sum of these age-specific incidence rates, with weights corresponding to the proportion of each age group in the 2011 standard population (24). Incidence per 10,000 hospital admissions was also calculated for the period of 2011–2012 to 2021–2022. Patient admissions data were obtained from the Australian Institute of Health and Welfare website for the financial years 2011–2012 to 2021–2022 (25).

Antimicrobial resistance was calculated as the annual proportion of resistant isolates among all tested isolates. To assess changes over time, we applied the Cochran-Armitage test for trends in proportions. A *P* value of 0.05 was considered statistically significant. All analyses were performed using R version 4.4.1.

## RESULTS

**Fig 1:**
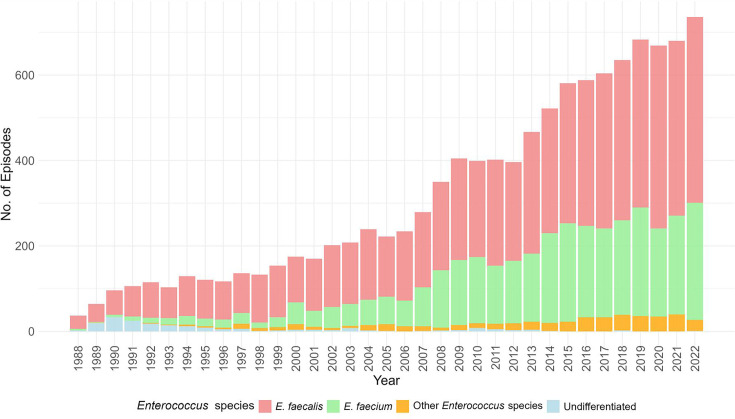
Number of enterococcal bacteremia episodes, per species and year, 1988–2022, Victoria, Australia.

A total of 11,157 unique enterococcal bacteremia episodes were identified among 10,269 patients from July 1988 to December 2022. The number of episodes per year is shown in Fig. 1. There were 11 *Enterococcus* species identified, the majority of isolates 61.9% (*n* = 6,915) were *E. faecalis* and 31.9% (*n* = 3,558) *E. faecium,* while the remaining were *Enterococcus casseliflavus* (*n* = 137), *Enterococcus gallinarum* (*n* = 130), *Enterococcus avium* (*n* = 97), *Enterococcus durans* (*n* = 41), *Enterococcus hirae* (*n* = 36), *Enterococcus raffinosus* (*n* = 34), *Enterococcus cecrorum* (*n* = 5), *Enterococcus mundtii* (*n* = 2), and *Enterococcus saccharolyticus* (*n* = 1). The remaining 199 episodes were reported as “species,” which we classified as undifferentiated. Most of these isolates were historical, mainly reported during the 1990s (Fig. 1).

**Fig 2:**
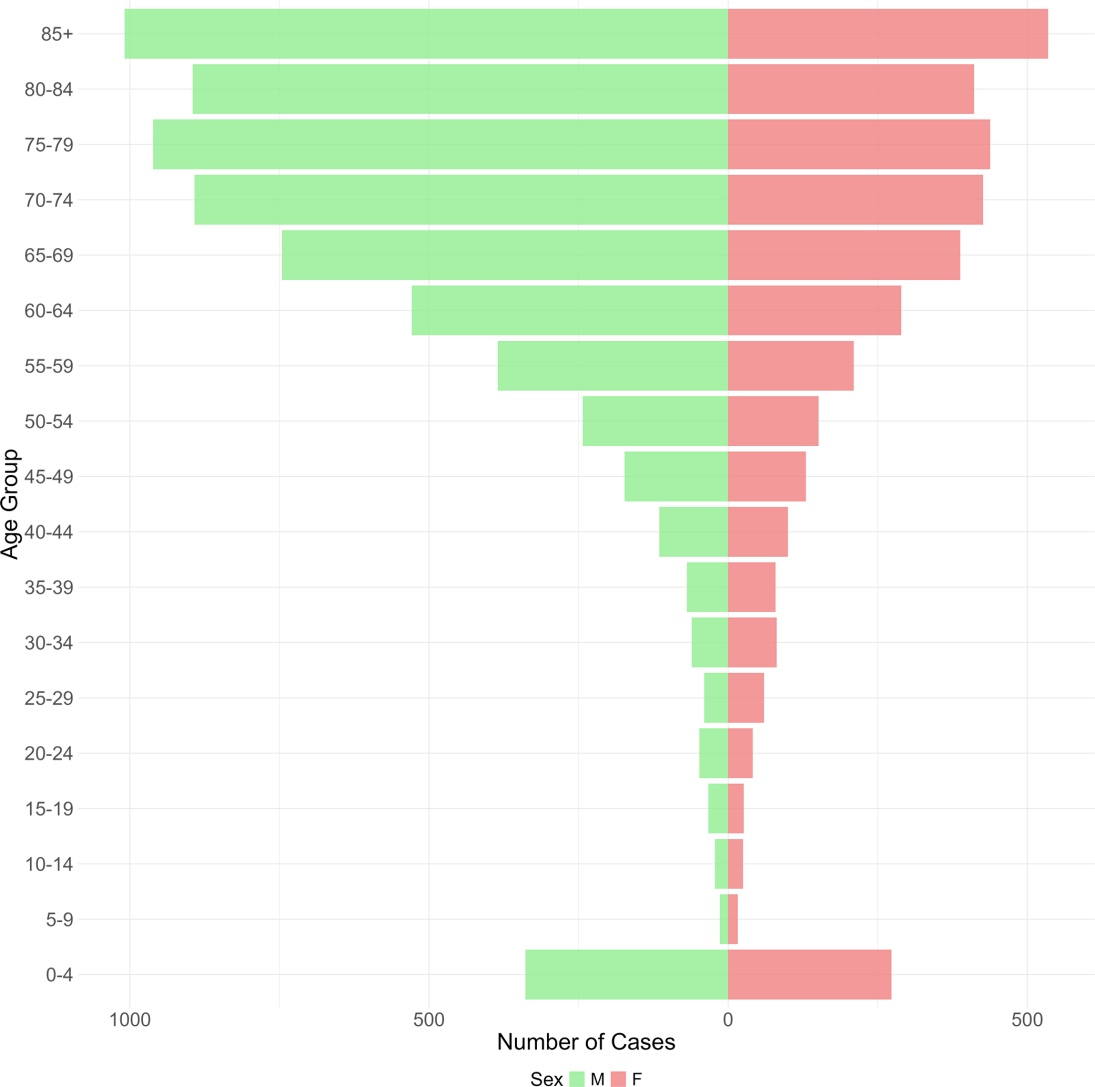
Age and sex distribution of patients with enterococcal bacteremia, 1988–2022, Victoria, Australia.

The median age of patients was 71, with those aged ≥60 (*n* = 7,519, 73.2%) and males (*n* = 6,583, 64.0%) most frequently represented among the patient cohort (Fig. 2). Among pediatric patients, ages 0–4 years constitute the largest group (Fig. 2).

**Fig 3:**
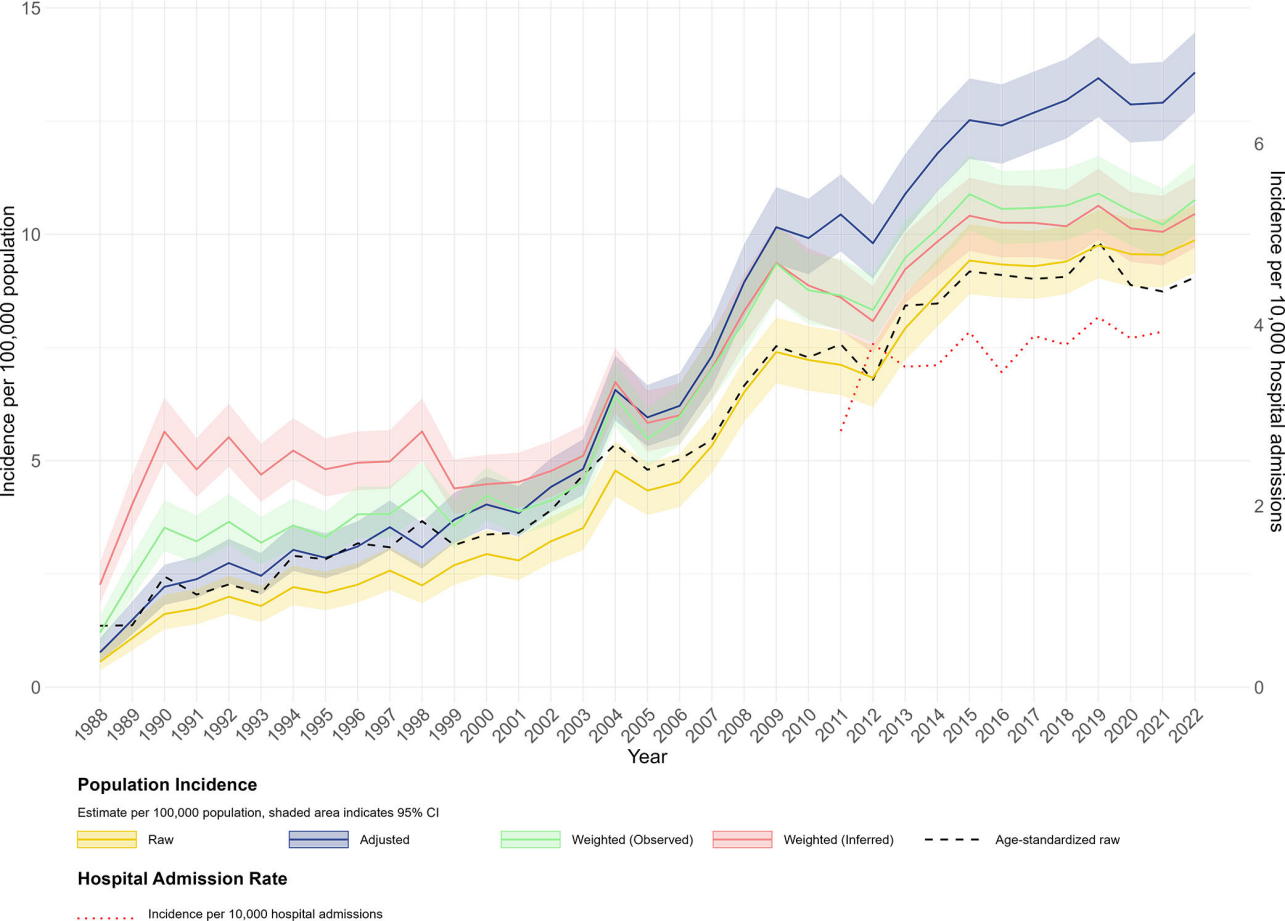
Annual incidence trends of enterococcal bacteremia in Victoria, Australia, using denominators of population (1988-2022) or hospital admission (2011-2021). Incidence estimates are presented per 100,000 population, raw incidence (yellow), hospital coverage adjusted (dark blue), IPW weighted incidence based on Observed participation (green) and inferred participation (pink) with shaded area indicating 95% confidence intervals. Age-standardized incidence is shown as a dashed black line. Hospital admission incidence rate per 10,000 hospital admissions is shown as a red dotted line.

The adjusted and weighted population-based incidence of enterococcal bacteremia in Victoria increased almost threefold over the 35-year study period, rising from fewer than 3 episodes per 100,000 population in 1988 to more than 10 per 10,000 by 2022 (Fig. 3). This upward trend was consistent across all estimation methods. In earlier years, weighted incidence estimates were higher than raw and adjusted incidence estimates. We also found that age-standardized incidence was similar to raw incidence rates. Similarly, the incidence per 10,000 hospital admissions increased over the period for which data are available (2011–2022), ranging between 2.8 and 4.0 (Fig. 3).

**Fig 4:**
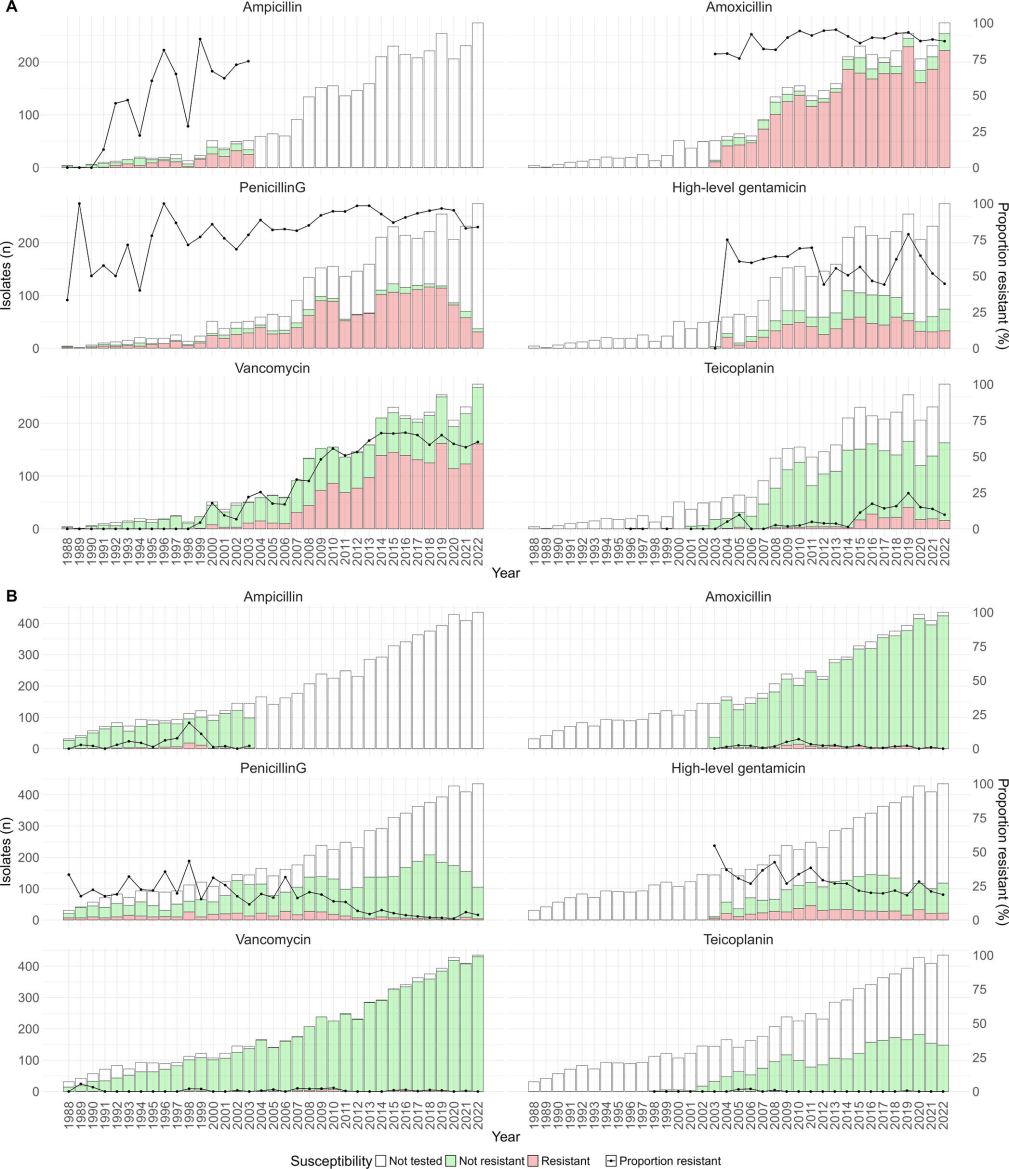
Resistance proportion to selected antibiotics among (**A**). *E. faecium and* (**B**). *E. faecalis* isolates from 1988 to 2022, Victoria, Australia.

Among *E. faecium* isolates, high proportions of resistance were observed to β-lactam class antibiotics, including ampicillin, amoxicillin, and penicillin G, all showing an overall increasing trend (Cochran-Armitage test; *P*-value < 0.0001 for ampicillin, *P*-value = 0.04 for amoxicillin, and *P*-value < 0.0001 for penicillin G) (Fig. 4A). Additionally, vancomycin resistance proportions significantly increased (Cochran-Armitage test; *P*-value < 0.0001) during the study period, fluctuating between 50.7% to 66.5% from 2011 to 2022. The proportion of *E. faecium* isolates resistant to teicoplanin also increased significantly (*P*-value < 0.0001). This sharp increase was first observed between 2014 and 2015, rising from 1.3% to 11.3% and peaking at 24.7% in 2019. By contrast, a decreasing trend was observed in the resistance proportions to high-level gentamicin (*P*-value = 0.027). Daptomycin and linezolid resistance proportions, while fluctuating, remained below 4% during the study period (Fig. S2A).

We found that while *E. faecalis* isolates were generally sensitive to most tested antimicrobials; they exhibited higher resistance to penicillin G and high-level gentamicin compared to other antimicrobials (Fig. 4B). Interestingly, these resistance proportions have decreased throughout the study (*P* value < 0.05). We initially observed daptomycin resistance proportions of 12.3% (12/97) in 2021 and 19.1% (18/94) in 2022, based on VITEK results reported by the submitting laboratory. Given these unusually elevated rates and the fact that they were all from the same laboratory, we requested a subset of 2022 isolates (13/18) for confirmatory testing with broth microdilution. Our results showed that all tested isolates were daptomycin susceptible according to CLSI guidelines (data not shown). To avoid misinterpretation, these data were excluded from further analysis. There was no significant trend in linezolid resistance (Fig. S2B).

## DISCUSSION

This study provides the most comprehensive longitudinal analysis of enterococcal bacteremia in Victoria, Australia, utilizing 35 years of surveillance data. We improved the representativeness of our data set by applying IPW to derive population-based estimates that more accurately reflect the underlying burden of enterococcal bacteremia in Victoria. Our analysis demonstrated a substantial increase in incidence in recent years, across all estimation methods, indicating a genuine rise in disease burden. Similarly, AMR trend analysis showed a sustained upward trend in vancomycin resistance among *E. faecium* isolates, reinforcing the clinical significance of this high-priority pathogen.

Over the study period, the most identified species from enterococcal bacteremia isolates analyzed in this study were *E. faecalis* and *E. faecium*, consistent with recent Australian reports (9). However, reports from other countries (26, 27) have indicated that *E. faecium* was the most prevalent species among BSI isolates. The shift toward *E. faecium* may be driven by factors such as excessive antibiotic use and its ability to acquire and spread resistance genes. These factors may change across different regions and patient populations (28). Our data also show that bacteremia episodes due to undifferentiated *Enterococcus* species were mostly observed in the 1990s, likely due to limitations in phenotypic identification methods at the time (29).

We found that males and patients aged 60 and over constitute the largest proportion of our patient population, a trend consistent with findings from other Australian states (30) and international studies (31–33). Genetic, hormonal, socioeconomic, and environmental factors are believed to contribute to these sex differences in susceptibility to infectious diseases, including vancomycin-resistant BSI (34).

All enterococcal bacteremia incidence estimates showed an overall upward trend. Statewide population-based incidence of enterococcal bacteremia in Victoria increased to over 10 episodes per 100,000 population by 2022, with hospital admissions-based incidence also increasing over the reported period (Fig. 3). Age-standardized trends closely mirrored the raw incidence estimates (Fig. 3), suggesting that population aging over the study period does not account for the increase observed. Although age structure did not explain the increase we observed, other factors may have contributed to this trend. During the study period and particularly in the mid-2000s to mid-2010s, the increase in incidence coincided with the introduction and availability of several microbiological and molecular technologies including polymerase chain reaction-based methods and later MALDI-TOF mass spectrometry. These technologies improved the speed and accuracy of *Enterococcus* species identification and may have contributed to better enterococcal bacteremia case ascertainment (35, 36).

Our incidence estimates are lower than the 19.9 per 100,000 person-years incidence rate reported in the Barwon region of Victoria between 2010 and 2017, the only other Victorian study available (37). This difference may reflect regional variation or more complete cases captured within a single healthcare network compared to our estimates, which were derived from voluntary statewide surveillance data.

Comparisons with global data are challenging due to differences in surveillance systems, data capture methods, and population demographics. Nevertheless, our incidence estimates are consistent with Canadian studies, which reported rates of 6.9 per 100,000 (2000–2008) and 10 per 100,000 (2011–2018), but remained lower than those reported in Denmark (19.6 per 100,000 between 2006 and 2009) and England where the national rates increased from 9.6 per 100,000 in 2012 to 15.9 per 100,000 in 2021 (26, 31, 32, 38).

The temporal patterns observed in our population-based modeled incidence estimates highlight the value of IPW to account for changes in participation over time. As shown in Fig. 3, the model predicted incidence estimates with observed and inferred participation were notably inflated in earlier years, especially between 1998 and 2001, compared to raw and adjusted estimates. This inflation reflects how the model assigns higher weights to hospitals within certain remoteness areas and peer groups (see supplementary methods, sections C.1 and C.2) that had lower participation probabilities. In later years, as hospital participation improved, lower weights (i.e., closer to 1.0) were given. When a hospital from an underrepresented group reported an episode in a year where its participation was unexpected, the episode count was upweighted to represent similar non-reporting hospitals, resulting in an overall higher incidence estimate.

Given the rationale underlying the IPW method, we consider IPW-based estimates to be more representative of our data because they account for variation in hospital participation. They offer a more robust alternative to crude raw counts, which underestimate incidence and to adjusted estimates, which may overestimate incidence by assuming a constant underreporting rate across time, location, and hospital types. Such uniform adjustments fail to account for real-world variation in surveillance data, potentially introducing more bias.

The application of IPW has been validated in previous studies as an effective method to improve estimates in similar contexts using data derived from incomplete or biased surveillance data. For example, Vicentini et al. have used IPW to correct for length-of-stay-biased sampling, resulting in more accurate incidence estimates from point prevalence surveillance data (39). Furthermore, a Belgian study has also used IPW to reduce laboratory participation bias in sentinel surveillance data (22).

Over the three decades of surveillance, the proportion of VREfm isolates showed an overall increasing trend, with consistently high rates over the last decade. Importantly, VREfm proportions in Victoria are much higher than those reported in other Australian states and global data (12, 40–42). Multiple factors have been previously linked to the increasing rates of vancomycin resistance, including patient colonization, environmental contamination within healthcare facilities, and inadequate infection control practices (43).

In 2015, we observed the first sharp increase in teicoplanin resistance, which coincided with a nationwide increase in *vanA*-harboring VREfm bacteremia isolates that reached 22% by 2016 (44). The *vanA* gene cluster is associated with resistance to both vancomycin and teicoplanin (45), suggesting that the increased prevalence of *vanA* VREfm likely contributed to the observed increase in teicoplanin resistance. Furthermore, the first outbreak of *vanA* VREfm in Victoria was also reported in 2015 in a hospital with endemic *vanB* VRE (15). In contrast, the proportion of *E. faecium* isolates resistant to high-level gentamicin showed a decreasing trend, consistent with national trends during the same period (12).

While our study observed low rates of daptomycin resistance among BSI isolates, other Victorian studies reported substantially higher rates. Across all VREfm isolates collected in 2015, 2017, and 2018, an overall rate of daptomycin resistance was 19.4% (46). Notably, this study included all specimen types and *vanA*-harboring isolates, which were found to be more likely to exhibit reduced susceptibility to daptomycin in that setting. This may also reflect that invasive isolates are not always representative of resistance rates in non-invasive isolates (47). Another possibility is variation in laboratory testing and reporting practices, as some hospitals may not have routinely tested for or reported daptomycin resistance. As daptomycin and linezolid are the primary treatment options for VREfm, the emergence of isolates resistant to both vancomycin and daptomycin represents a significant clinical concern, potentially leaving linezolid as the only remaining therapeutic option.

Fortunately, *E. faecalis* isolates remained sensitive to the reported antibiotics throughout the study period. The observed decreasing trend in high-level gentamicin resistance among *E. faecalis* isolates is consistent with national trends (12). In contrast, higher levels of resistance to vancomycin, linezolid, and gentamicin have been reported in other regions including Southeast Asia, Eastern Mediterranean, and African countries. These patterns were highlighted in a global meta-analysis of AMR in *E. faecalis* bloodstream isolates between 2000 and 2018. Limited resources in some settings can pose challenges in maintaining good hygiene, adequate infection control, and antimicrobial stewardship programs (48).

Our study had some limitations. First, participation in the VHPSS database is voluntary, which means that not all laboratories in Victoria report observed cases, and even among participating laboratories, case reporting may not be consistent over time, limiting representativeness. Second, determining hospital participation across such a long period (35 years) was challenging, particularly given the large number of hospitals and variability in their reporting patterns. Although we applied IPW to adjust for variation in hospital participation, data should be interpreted carefully as the model assumes that data are missing at random, which may not be fully met in this case. Third, inferred participation required assumptions based on institutional knowledge and was not always verifiable through data only. Fourth, the limited availability of publicly accessible hospital attributes may have affected the accuracy of participation modeling and consequently our estimated IPW-based weights. Fourth, the lack of molecular data on isolates hinders the identification of VRE genotypes or AMR mechanisms that could have been associated with the observed increase in VRE. Fifth, it is important to consider when interpreting AST data that laboratories varied in AST methods and international standards used (CLSI vs EUCAST), breakpoints changed over time, and only categorical interpretations with no MIC values were available, limiting our ability to apply consistent interpretive criteria across reporting periods. Finally, similar to other surveillance data sets, clinical data were not captured by the VHPSS database; therefore, we could not assess clinical impact. Future studies could focus on linking clinical outcome data to identify risk factors within the Victorian population and potentially incorporating genomic surveillance of BSI isolates to improve our understanding of the drivers of this increase.

In summary, our study findings highlight the escalating population-based incidence of enterococcal bacteremia and the persistently high rates of VREfm in Victoria. These increasing resistance levels reinforce the urgent need for improved surveillance, infection control measures, and antimicrobial stewardship. To further strengthen the surveillance quality of the VHPSS, we propose conducting annual audits of selected non-participating hospitals to identify barriers to reporting and implement strategies to enhance engagement and data completeness. By applying IPW, we improved the representativeness of incidence estimates derived from a voluntary surveillance system, showcasing this as a framework for the future application of IPW to improve incidence estimation of other conditions reported to VHPSS and for similar surveillance systems around the world.

## SUPPLEMENTAL MATERIAL

Supplemental material aac.01526-25-s0001.docx Supplemental methods; Fig. S1 and S2.

ASM does not own the copyrights to Supplemental Material that may be linked to, or accessed through, an article. The authors have granted ASM a non-exclusive, world-wide license to publish the Supplemental Material files. Please contact the corresponding author directly for reuse.
