# Supplementary material for: Rising burden of enterococcal bacteremia in Victoria, Australia: population-based incidence and antimicrobial resistance trends from three decades of surveillance
Source: Antimicrob Agents Chemother. 2026 Feb 4;70(3):e01526-25. doi: 10.1128/aac.01526-25 (PMC12959146; doi:10.1128/aac.01526-25)
Supplement: Supplemental material — Supplemental methods; Fig. S1 and S2. [file aac.01526-25-s0001.docx]

**Table of Contents**

[Supplementary methods: 2](#_Toc206786723)

[A. Datasets and data preparation 2](#_Toc206786724)

[A.1 Enterococcal specific dataset 2](#_Toc206786725)

[A.2 All pathogens dataset 2](#_Toc206786726)

[B. Hospital participation definitions 2](#_Toc206786727)

[B.1 Observed (data- driven) participation 2](#_Toc206786728)

[B.2 Inferred participation 2](#_Toc206786729)

[C. Hospital characteristics 4](#_Toc206786730)

[C.1 Peer group 4](#_Toc206786731)

[C.2 Remoteness area 4](#_Toc206786732)

[D. Propensity score modelling using Generalized Linear models (GLM) 4](#_Toc206786733)

[D.1 Inverse probability weighting strategy 4](#_Toc206786734)

[E. Estimation of raw, adjusted and weighted incidence 4](#_Toc206786735)

[E.1 Raw incidence 4](#_Toc206786736)

[E.2 Coverage adjusted incidence 4](#_Toc206786737)

[E.3 Weighted incidence 5](#_Toc206786738)

[References 5](#_Toc206786739)

[Supplementary figures: 6](#_Toc206786740)

[Supplementary Figure 1 6](#_Toc206786741)

[Supplementary Figure 2 7](#_Toc206786742)

## Supplementary methods:

### Datasets and data preparation

Two main datasets were used:

A.1 Enterococcal specific dataset

This dataset includes all reports of bacteraemia caused by *Enterococcus species* (n=11,157). Records were excluded if hospital information was missing (n=623) or if the hospital lacked classification data (n=55). This cleaned dataset was used to generate annual case counts by hospital.

A.2 All pathogens dataset

This broader dataset includes all bacteraemia episodes reported to VHPSS regardless of pathogen. It was used to derive hospital participation status.

From these datasets, we constructed a comprehensive dataset (enhanced_cases_data) consisting of all (n= 179) hospital-year combinations between 1988 and 2022, regardless of whether any episodes were reported. Each row of this dataset represents a unique combination of hospital and year, including the number of reported enterococcal bacteraemia (Cases_Reported_ent), total bacteraemia episodes by any pathogen (Cases_Reported) and associated hospital characteristics (remoteness area and peer group). Two binary indicators of participation were also added, observed and inferred participation.

### Hospital participation definitions

We determined hospital participation based on two definitions:

B.1 Observed (data- driven) participation

A hospital was considered a participant in a given year if it reported any episode of bacteraemia caused by any pathogen.

B.2 Inferred participation

Based on institutional knowledge about historical patterns of hospital engagement, we modified the observed participation based institutional knowledge or expected hospital ability to report. This definition aims to distinguish between true zero episode counts and potential underreporting due to lack of data submission. Participation was informed by hospital peer groups and reporting history as summarized in table 1.

For each year from 1988 to 2022, hospitals were assigned an indicator of 1 if they were considered to have participated and an indicator of 0 for years in which they did not. Each participation definition was tested in a separate IPW model.

| **Peer group** | **Inferred participation rule** |
| --- | --- |
| Public acute group B & C, Private acute group C & D, Mixed sub- & non-acute hospitals | Applied 3-year rule: participation = 1 if reported more than a case in any 3-year window. |
| Public acute group D hospitals | Participation = 1 from the first year they participated onwards |
| Principal referral hospitals | St Vincent’s and Royal Melbourne hospital (city campus): Participation = 0 from 1996 onwards  Austin hospital: Participation =0 from 1998 onwards |
| Private acute group A hospitals | Epworth Hospital [Richmond], The Valley Private Hospital [Mulgrave], Epworth Freemasons: Participation = 0 from 2003 onward |
| Private acute group B hospitals | Epworth Eastern Hospital: Participation = 0 all years.  St Vincent’s Private Hospital [Fitzroy]: Participation = 0 before 2018.  Others: Participation = 1 from first year they participate onward |
| Women’s hospitals | Frances Perry Private Hospital [Carlton]: Participation = 1 for all years |
| Very small hospitals | Participation = 1 from 2004 onward. |
| Public acute group A and Children’s hospitals | No rule applied (unchanged *) |
| Public rehabilitation hospitals, Other acute specialised hospitals, Private rehabilitation hospitals. Drug & alcohol hospitals, Mixed day procedure hospitals, Dialysis clinics, Unpeered hospitals, Gynaecology day hospitals, Endoscopy centres, Private acute psychiatric hospitals, Oral & maxillofacial surgery centres, Haematology and oncology clinics. | Participation =1 in all years |

*Unchanged: The participation indicator for these hospitals was based solely on observed episodes and was not altered by additional inference rules.

Table 1. Participation assignment rules by hospital peer groups.

### Hospital characteristics

Two hospital specific characteristics were obtained from the Australian institute of health and welfare (AIHW) and used as predictors of participation:

C.1 Peer group

A categorical grouping of hospitals based on the type of services they provide.

C.2 Remoteness area

A geographic classification based on location and accessibility to healthcare services, categorized as major city, inner regional and outer regional.

To avoid collinearity between these variables, we combined them into a single variable (Remoteness_PeerGroup).

### Propensity score modelling using Generalized Linear models (GLM)

To estimate probability of hospital participation, we used logistic regression (GLM with binomial family) with hospital characteristics and year as predictors. the glm() and predict() functions from the R stats package (version 4.4.1) were used to fit the model and calculate the propensity scores.

D.1 Inverse probability weighting strategy

In alignment with the Horvitz-Thompson estimator ^[1]^, the weight was calculated as the inverse of a hospital’s likelihood of participation (1/ propensity score). Non-participating hospitals were assigned a weight of zero.

### Estimation of raw, adjusted and weighted incidence

The population of Victoria per year was obtained from the Australian Bureau of Statistics website and was used as the denominator in the following analyses.

Incidence per 100,000 of Victorian population was calculated for each year from 1988 to 2022 as:

E.1 Raw incidence = $\frac{Total number of Enterococcal bacteremia episodes}{Population of victoria} \times100,000$

E.2 Coverage adjusted incidence = $\frac{Raw incidence}{\% Covered}$

%Covered: Estimated proportion of the population served by participating hospitals.

This was calculated for the time period 2011-12 to 2021-22 by

$\frac{Total number of admissions of hospitals that reported episodes to VHPSS}{Total hospital admissions in Victoria}$

For the remaining years, we used the mean % covered, which was 73%.

E.3 Weighted incidence:

Then, to calculate the weighted number of episodes for each hospital and year combination:

weighted number of episodes per hospital-year = Episodes of enterococcal bacteraemia X weight

Weighted incidence = $\frac{Sum of weighted number of episodes}{Population of victoria}\times100,000$

### References

1. Horvitz, D. G., & and Thompson, D. J. (1952). A Generalization of Sampling Without Replacement from a Finite Universe. *Journal of the American Statistical Association*, *47*(260), 663–685. https://doi.org/10.1080/01621459.1952.10483446

## Supplementary figures


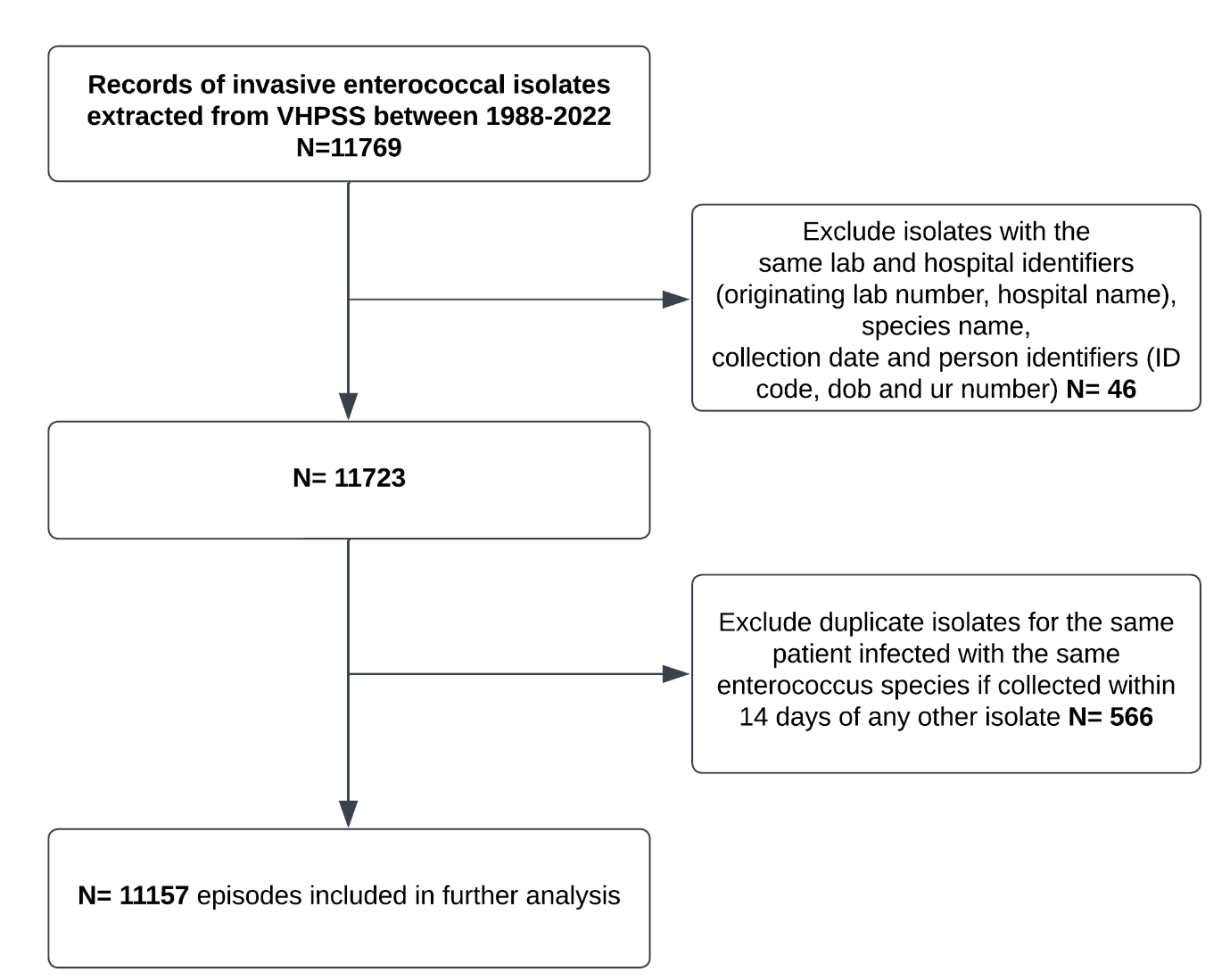


Supplementary Figure 1. Exclusion criteria of invasive enterococcal isolates.


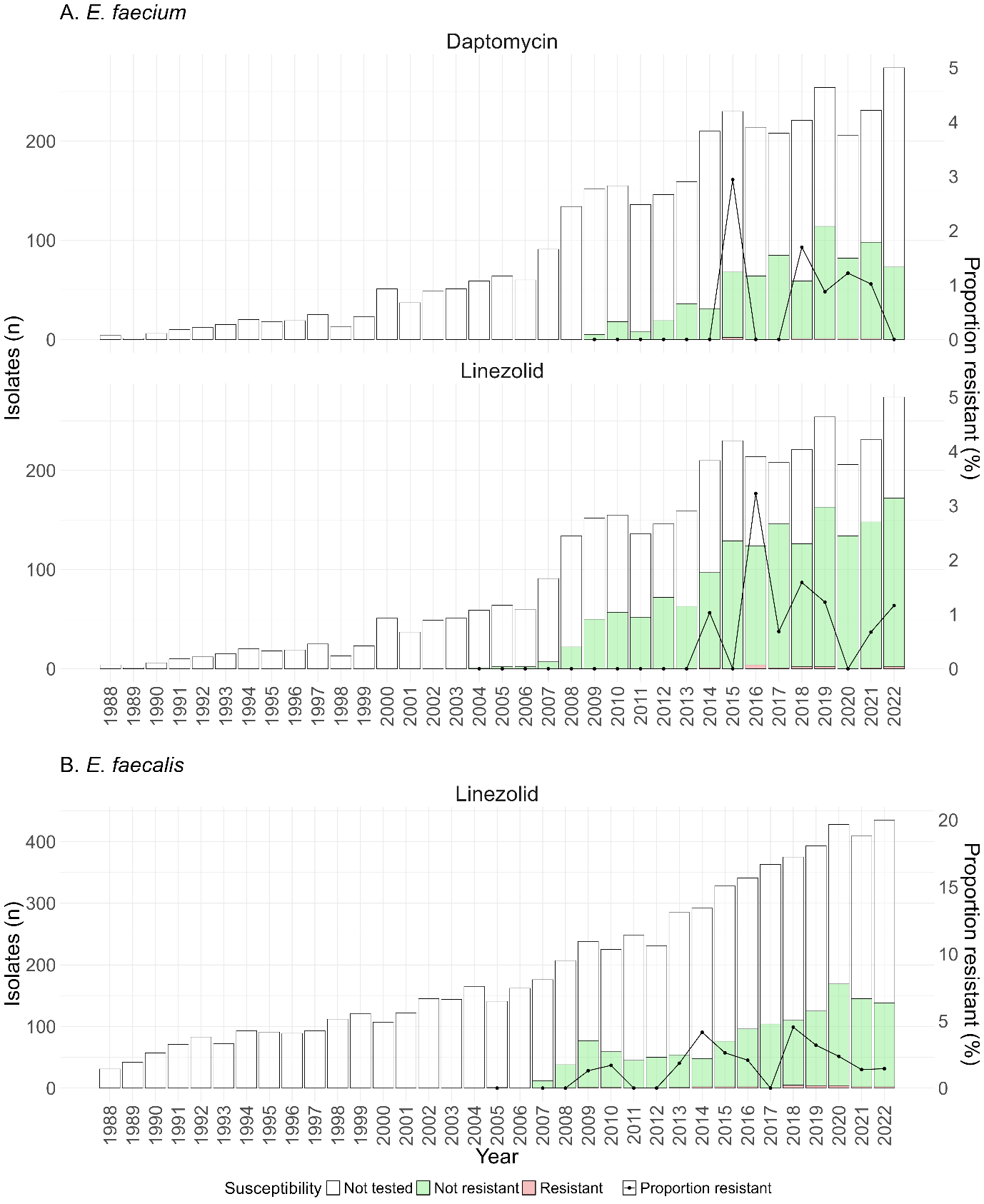
Supplementary Figure 2. Resistance proportion to daptomycin and/or linezolid among *E. faecium* and *E. faecalis* isolates from 1988 to 2022. **A**) *E. faecium* isolates **B**) *E. faecalis* isolates. Note that secondary y axis scales differ between A and B.
